# Assessing biomarkers and neuropsychological outcomes in rural populations exposed to organophosphate pesticides in Chile – study design and protocol

**DOI:** 10.1186/s12889-015-1463-5

**Published:** 2015-02-10

**Authors:** Muriel Ramírez-Santana, Liliana Zúñiga, Sebastián Corral, Rodrigo Sandoval, Paul TJ Scheepers, Koos Van der Velden, Nel Roeleveld, Floria Pancetti

**Affiliations:** Department of Public Health, Faculty of Medicine, Universidad Católica del Norte, Calle Larrondo 1281, Postal Code 1780000 Coquimbo, Chile; Department of Biomedical Sciences, Faculty of Medicine, Universidad Católica del Norte, Calle Larrondo 1281, Postal Code 1780000 Coquimbo, Chile; Psychology Department, FACSO, Universidad de Chile, Santiago, Chile; Department for Health Evidence, Radboud Institute for Health Sciences, Radboud university medical center, Geert Grooteplein-Zuid 10, 6525 GA Nijmegen, The Netherlands; Department of Primary and Community Care, Radboud Institute for Health Sciences, Radboud university medical center, Geert Grooteplein-Zuid 10, 6525 GA Nijmegen, The Netherlands; Department of Pedatrics, Radboudumc Amalia Children’s Hospital, Radboud university medical center, Geert Grooteplein-Zuid 10, 6525 GA Nijmegen, The Netherlands

**Keywords:** Pesticides, Biomarker, Chronic exposure

## Abstract

**Background:**

Health effects of pesticides are easily diagnosed when acute poisonings occurs, nevertheless, consequences from chronic exposure can only be observed when neuropsychiatric, neurodegenerative or oncologic pathologies appear. Therefore, early monitoring of this type of exposure is especially relevant to avoid the consequences of pathologies previously described; especially concerning workers exposed to pesticides on the job. For acute organophosphate pesticides (OPP) exposure, two biomarkers have been validated: plasma cholinesterase (ChE) and acetylcholinesterase (AChE) from erythrocytes. These enzymes become inhibited when people are exposed to high doses of organophosphate pesticides, along with clear signs and symptoms of acute poisoning; therefore, they do not serve to identify risk from chronic exposure. This study aims to assess a novel biomarker that could reflect neuropsychological deterioration associated with long-term exposure to organophosphate pesticides via the enzyme acylpeptide-hydrolase (ACPH), which has been recently identified as a direct target of action for some organophosphate compounds.

**Methods/Design:**

Three population groups were recruited during three years (2011–2013): Group I having no exposure to pesticides, which included people living in Chilean coastal areas far from farms (external control); Group II included those individuals living within the rural and farming area (internal control) but not occupationally exposed to pesticides; and Group III living in rural areas, employed in agricultural labour and having had direct contact with pesticides for more than five years. Blood samples to assess biomarkers were taken and neuropsychological evaluations carried out seasonally; in three time frames for the occupationally exposed group (before, during and after fumigation period); in two time frames for internal control group (before and during fumigation), and only once for the external controls. Neuropsychological evaluations considered cognitive functions, affectivity and psychomotor activity. The biomarkers measured included ChE, AChE and ACPH. Statistical analysis and mathematical modelling used both laboratory results and neuropsychological testing outcomes in order to assess whether ACPH would be acceptable as biomarker for chronic exposure to OPP.

**Discussion:**

This study protocol has been implemented successfully during the time frames mentioned above for seasons 2011, 2012 and 2013–2014.

## Background

Human exposure to organophosphate pesticides (OPP) have been extensively documented showing health problems, associated primarily with agricultural workers having occupational exposure in developing countries [1]. While acute poisonings are relatively easy to diagnose because they are accompanied with symptoms of cholinergic overstimulation [2], the effects of chronic, long-term exposure to low OPP doses only become evident when carcinogenic, teratogenic [3,4] or neurodegenerative pathologies appear [5-7]. The nervous system is particularly sensitive to the effects of OPP, therefore early bio-monitoring of neurotoxic effects in exposed people can prevent the onset of future neurodegenerative diseases by taking some measures to avoid or diminish the level of OPP exposure.

The diagnosis of acute or chronic exposure to organophosphate pesticides (OPP) usually employs two different blood enzymes as biomarkers; plasma pseudocholinesterase (or butyrylcholinesterase, BuChE) and erythrocyte acetylcholinesterase (AChE), the later being the enzyme most used for estimating chronic exposure [8]. The catalytic activity of both these enzymes is inhibited by OPP and in the case of AChE inhibition, where the enzyme is expressed in the synapses of the nervous system, this inhibition reflects cholinergic overstimulation responsible for the signs and symptoms of OPP poisoning. Therefore, their usefulness as biomarkers of low-dose exposure to OPP is limited. Because of this, it is necessary to develop a more sensitive blood biomarker that account for long-term, low-dose exposure to OPP.

There is much evidence relating low-level and prolonged OPP exposure with cognitive performance deterioration. Scientific literature reporting the effects of long-term exposure to OPP in cognitive processes strongly indicates that the impairment of cognitive or neurological processes correlates with the time of exposure to OPP [1,2]. Rohlman and collaborators [8] indicate that the appearance of this disorder does not always correlate with an inhibited cholinesterase activity, suggesting that the action of OPP depends on the type and burden of pesticides to which people are exposed. At the same time, it is important to mention that most studies have measured biomarkers and neuropsychological performance only once, not considering different fumigation seasons that allow for the possibility of reversibility on neuropsychological performance [9,10]. Methodological weaknesses of previous studies are related to examining different occupational groups with different levels and routes of exposure, having different time periods, low samples, and other epidemiological constraints that limit variables of exposure and health effects, among others [11]. Furthermore, existing biomarkers are not that sensitive and do not allow for measuring chronic exposure nor chronic effects [8].

Acylpeptide hydrolase (ACPH) is a non-cholinesterase target of OPP that seems to be involved in the effects of these molecules have on cognitive processes [12]. ACPH, also known as acylamino-acid releasing enzyme or acylaminoacyl peptidase, is a homomeric tetramer that belongs to the family of prolyl-oligopeptidase of the serine hydrolases [13] and catalyzes the hydrolysis of several peptides possessing an acylated N-terminal amino acid to generate an acylated amino acid and a free N-terminal peptide [14,15]. It has also been described as a truncated form of the enzyme having endopeptidase activity [16]. In mammals, ACPH acts in coordination with proteasome to clear cytotoxic denatured proteins from cells [17,18]. Strong inhibition of ACPH activity leads to apoptosis [19], and deletions in the gene encoding ACPH leading to deficiencies of this enzyme have been observed in renal and small-cell lung carcinomas [20,21]. Regarding the role of ACPH in the nervous system, it is known that ACPH is involved in the moderation of synaptic activity [22] and can be found localized in pre-synaptic compartments of the rat telencephalon [23]. Interestingly, it has been reported that ACPH can degrade monomers, dimers and trimers of the Aβ_1–40_ peptide [24,25].

Richards and collaborators reported that some OPP such as chlorpyrifos-methyl oxon, dichlorvos, and diisopropyl fluorophosphate (DFP) exhibit a higher affinity toward ACPH compared to AChE. Specifically, dichlorvos and DFP showed an increased affinity of 6.6 - 10.6 fold toward ACPH with respect to AChE [26]. On the other hand, it has been demonstrated in animal models that the inhibition of ACPH by the OPP dichlorvos had biphasic effects in the cellular mechanisms responsible for learning and memory, while low doses of dichlorvos had positive effects on synaptic plasticity processes, and high doses or prolonged exposure times have the opposite effect and are neurotoxic [12]. In spite of this, it has been described that chlorpiryfos-oxon, diazoxon, paraoxon and mipafox, among other organophosphate compounds, inhibit ACPH as well as AChE activity from erythrocytes. This lack of specificity is compensated for by the persistence of inhibition toward ACPH activity (more than four days) compared with the inhibition of erythrocyte AChE activity or plasma ChE activity, which has a half- life of 11 days [27,28].

These findings support the notion of the usefulness of erythrocyte ACPH activity as having high sensibility and being a reliable biomarker for monitoring chronic exposure to OPP [12] associated with cognitive deterioration.

### Aims and objectives

The main purpose of this study is to develop measurement of ACPH activity as novel erythrocyte biomarkers that will help identify early diagnosis of chronic exposure to OPP associated with neuropsychological impairment. The study design addresses some of the methodological concerns described by previous research [10,11,29]: the evaluation of more than one control group and measuring biomarker activity along with neuropsychological performance at different moments during the spraying season (before, during and after spraying with pesticides).

### Specific objectives

- ▪ To obtain activity profiles of the two blood enzyme biomarkers commonly measured. We used AChE and ChE and the new biomarker, ACPH, in three cohorts with different levels of exposure to OPP: occupational, environmental and with no known exposure.
- ▪ To obtain the neuropsychological performance profiles in the three cohorts described above and to assess the risk of cognitive impairment in these populations.
- ▪ To correlate the enzymatic activities of each of the three biomarkers with the cognitive status in the cohorts described above with different levels of OPP exposure.
- ▪ To analyse changes in the enzyme activities and/or in cognitive performances within occupationally and environmentally exposed cohorts that are dependent upon the fumigation period (before, during and after fumigation).
- ▪ To establish if ACPH activity is a suitable biomarker of long-term exposure to OPP associated with cognitive deterioration.

## Methods/Design

### Settings and target population

The study was conducted between the fall of 2011 and the fall of 2014 in urban and rural locations of Coquimbo Region, in northern Chile. People from two urban locations (the cities of Coquimbo and La Serena) and four rural districts (La Higuera, Paihuano, Vicuña and Monte Patria) were recruited. The main agricultural activity is located at Paihuano, Vicuña and Monte Patria districts and is related to grapes and citrus farming. To be included in the study, the subjects had to fit the following criteria: between 18 and 50 years old, right-handed and without diagnosis of neurological or psychiatric illness. Three population groups were considered: Group I (External Control) individuals without environmental or occupational exposure to OPP and living in coastal locations; Group II (Internal Control) living in rural locations near farming activities and probably under environmental exposure; and Group III (Occupational Control) people occupationally exposed to pesticides and composed of agricultural labourers living in rural areas in direct contact with pesticides for more than 5 years. Within this third group there were blenders, fumigators, tractor drivers, supervisors, collectors and packing workers. Also, to be included in this group, individuals must never have suffered acute intoxication due to OPP.

**Figure 1:**
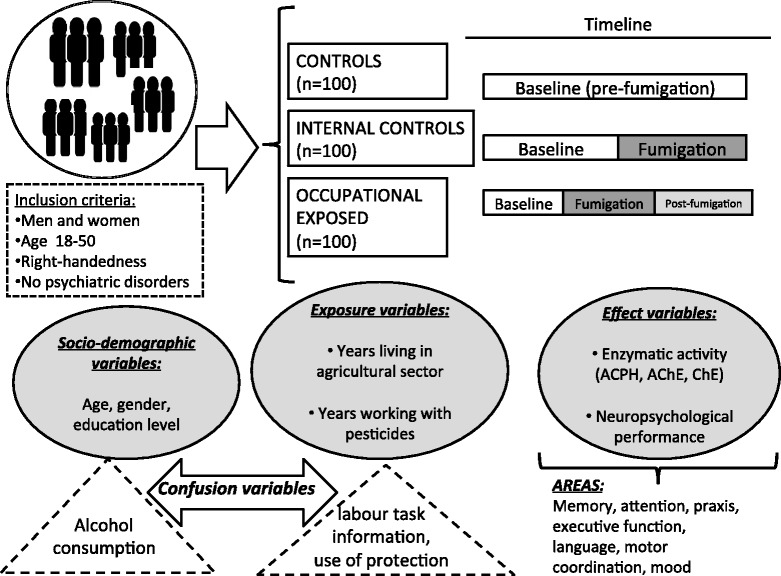
**Methodology chart: selected population groups, timeline evaluation and variables to measure.**

For all recruited individuals across the study, a baseline neuropsychological interview was done and a blood sample was taken. All procedures were accomplished in a mobile laboratory (an adapted Peugeot Boxer van, stationed permanently at the farms). All evaluations were carried out annually in three time frames for the occupationally exposed group (before, during and after fumigation period); in two time frames for internal control group (before and during fumigation) and a single time frame for the external controls. Exclusion criteria were: left-handedness, diagnosis of medical or psychiatric disease or disability and use of psychopharmacologic meds. For details, see Figure 1.

### Ethical considerations

Ethical approval for the study was obtained from the Research Ethics Committee of the Universidad Católica del Norte (The Catholic University of the North, in Coquimbo, Chile), dated August 25, 2014. Informed consent was explained to voluntary participants and signed by them before their recruitment.

### Power and sample size estimation

Two main objectives of the study were considered when calculating the number of participants to be recruited: a prevalence study (measuring enzymes activity and neuropsychological performance) and case control study (assessing ACPH as diagnostic test). The size of the occupationally exposed population in Coquimbo Region is 14,000 workers according to Ministry of Agriculture (2008) [30]. Considering that 10% of those workers have tasks involving direct use of pesticides (mixers, blenders, applicators), with a 95% exposure rate, a minimal sample size of 70 people was considered to be adequate for the prevalence study with 5% margin of error [31]. Secondly, for assessing the diagnostic test, the sample size was calculated based on the following equation [32]:

$$ n=\frac{{\left[{Z}_{\alpha}\ast \sqrt{2p\left(1-p\right)}\kern0.5em +{Z}_{\beta}\ast \sqrt{p_1\left(1-{p}_1\right)+{p}_2\left(1-{p}_2\right)}\right]}^2}{{\left({p}_1-{p}_2\right)}^2} $$

This considers the diagnostic test assessment as a case–control study; the “cases” being those people having neuropsychological impairment, and the “controls” being those people considered “normal” in their neuropsychological performance. Occupationally exposed people were expected to have less ACPH activity than non-occupationally exposed people. Assuming that the ACPH activity is oriented to have high sensitivity (0.999) for non-exposed people and have 60% specificity for non-exposed people, the “n” for this scenario would be 77 individuals. Finally, considering that between 20 to 30 per cent of the volunteers could be lost to yearly follow-up, the minimum number of study participants recruited were 100 people per group.

### Identification and recruitment of participants

Several meetings were held in different locations, where the field team explained the project and collected personal contact information from potential participants. Trained medical students employed a short questionnaire in order to eliminate people who did not fill full the enrolment criteria. The main questionnaire was then applied to those who met the requirements. A code was given to each participant, which was used for identification in the questionnaire, labelling blood samples and for neuropsychological tests results. The neuropsychological evaluation and blood samples were taken at the same time on a fixed date in agreement with the volunteers.

### Biomarkers evaluation

In blood samples, the levels of three enzymes related to OPP exposure in the three population groups were assessed. The evaluations were done in the field, in a mobile laboratory especially equipped for this purpose. The enzymes considered were: plasmatic AChE, ChE and ACPH; and were measured as explained in the “Setting and Target Population” section.

### Blood samples collection, storage, and transportation

A robust sampling and tracking system has was implemented to ensure both proper blood sample collection and survey data from each volunteer. Volunteers signed the consent form at the beginning of the process, after a detailed explanation of the project. Blood samples were collected by venepuncture in EDTA anticoagulant vacutainers, coded using a unique sample identification code and processed daily within 12 hours, keeping them at 4°C inside the mobile laboratory. Processing samples in the field consisted of separating plasma from cells by centrifugation (10 min, 3000 rpm, 4°C), after which the cell fraction was washed twice with cold PBS 1X and finally, each fraction (cells and plasma) was aliquot sequenced in three cryovial tubes labelled and frozen using liquid nitrogen. During transportation, the samples remained at −80°C until final analysis. The paramedic transporting and transferring all biological material to the technician kept a registration log of all collected specimens. Once in the lab, the technician checked and stored the samples.

### Laboratory analysis

One aliquot of each faction was thawed before enzymatic measurement. All determinations were done in triplicate based on Ellman *et al.,* (1961) method [33]. We used the spectrophotometer Specord type 205 (AnalitikaJena).

Plasmatic Cholinesterase (ChE) was measured directly using non-diluted plasma. Enzymatic activity was normalized using the protein content in the assay, protein content was determined by the bicinchoninic acid method [34] and enzymatic activity from each fraction was expressed as mean ± SD.

Erythrocytic Cholinesterase (AChE): To obtain the protein from broken erythrocytes, cells were lysed using dythiothreitol (1 mM). After centrifuge (10000 rpm, 30 min, 4°C) the supernatant was separated from the pellet and kept on ice to ACHP measurement. The pellet was washed once with cold phosphate buffer (0.05 M) and then measured in a reaction assay mixture (1.037 mL), which consisted of 5.5′-dithio-bis-2-nitrobenzoic acid (DTNB [0.241 mM]), acetylthiocholine (A-s-choline [0.029 mM]), disodium phosphate buffer (Na_2_HPO_4_ [0.31 mM]) and potassium phosphate buffer (KH_2_PO_4_ [0.023 mM]). Hydrolysis rate of acetylthiocholine is followed as indicated above (see plasma cholinesterase).

Acyl peptide hydrolase (ACPH): The measurement was determined using Tris–HCl (7.4 pH [100 mM] with DTT [1 mM]. The reagent mixture (1.020 mL) consisted of N-acetyl-L-alanine *p*-nitroaniline (AANA [3,9 mM]), Tris–HCl buffer (7.4 pH [95.59 mM], dythiothreitol (DTT [0.96 mM]) and dymethylsulfoxide (DMSO [275.88 mM]. Hydrolysis rate of AANA was then followed spectrophotometrically by the formation of *p*-nitroanilide (ε_410_ = 8800 M^−1^ cm^−^1) and measured at 405 nm, at 37°C during 40 min. The enzymatic activity was normalized to the haemoglobin content in the original blood sample volume. Haemoglobin was then measured using the cyan-methaemoglobin method [35]. Briefly, blood samples were mixed with a solution containing ferricyanide and cyanide. Haemoglobin changing to cyan-methaemoglobin was then measured at 520 nm.

### Quality control

In order to validate the replicability of our results, the National Institute of Public Health (Santiago, Chile) supported the quality control for plasmatic and erythrocytic cholinesterase. A random subset of samples were sent to the laboratory of Occupational Toxicology at the Department of Occupational Health in the Institute of Public Health, where plasmatic and erythrocytic cholinesterase activities were determined according to Ellman’s method. The resulting data obtained at the Institute of Public Health were matched to the results of the same samples obtained in our laboratory and statistical comparison performed. This procedure was done for each of the cohorts being evaluated.

### Methods for neuropsychological evaluation

In order to diagnose cognitive impairment, a Speech Therapist performed a psychological interviews and neuropsychological battery of tests for each volunteer. This battery covered three areas: cognitive functions, mood and psychomotor activity. We considered these three areas because the accumulation of acetylcholine in the synaptic cleft continuously stimulates the cholinergic synapses, triggering diverse symptoms in the neuro-conduct, cognitive and neuro-muscle areas. Table 1 shows the different cognitive functions and the tests used for their evaluation [36-39]. The time frame for a complete and individual evaluation was about three hours. The effects of fatigue on level of cognitive performance was addressed by beginning each evaluation with those tests most sensitive to fatigue, such as attention span, time of reaction and speed of process [40,41]. Additionally, a rest interval was included during the process.

**Table 1:** **Cognitive functions and associated tests**

**Function**	**Neuropsychological tests**
Memory	Rey auditory verbal learning (memory phase)
	Benton visual retention (4 subtest)
	Logic memory (WMS) (2 subtest)
	Serial digits learning
Constructive Praxis	Rey complex figure (copy phase)
	WAIS Cubes
Executive Functions	Barcelona test (categories)
	London tower
	Wisconsin Cards classification
	Stroop Test
	Battery for frontal evaluation FAB
Language	Boston denomination BNT
Attention	Digit span
	D2 Test
	Trail making test
	Corsi board (Software)
Motor Function	Pardue pegboard test (4 subtest)
	MOART reaction and movement time panel (2 subtest)
	Finger tapping test (2 subtest)
Mood status	Beck BDI-II depression inventory
	Hamilton anxiety scale

The evaluations were all performed by a Speech Therapist trained and supervised by a board certified neuropsychologist; in this way, inter-assessor’s bias is eliminated. All procedures are carried out in the mobile laboratory. To avoid a learning gap effect, tests that had a low learning component were selected and carried out at intervals of at least two months. In relation to assessment instruments, these were selected according to the following criteria: age of participants, reading and writing skills and absence of severe sensory deficits. It is necessary to indicate that, given the number of tests being applied and the time it took, we evaluated only two to three individuals a day. A baseline manual for measuring level of performance was used for each original set of tests, and scores of the exposed populations compared with scores of the control population. We calculated neuropsychological results clustered by area (cognitive functions: memory, attention span, constructive praxis; executive functions: mood and psychomotor activity) and by the average score given by test for each area. A unique final score will be calculated for each individual and evaluation time.

### Exposure characterization

Trained medical students conducted interviews using a questionnaire to assess personal, medical, social and occupational conditions. This questionnaire covered a broad spectrum of information in order to avoid misperceptions, because most people involved in the study are farmers or fishermen with very basic educational levels. The different topics in the inquiry included: personal data (gender, age, occupation, address and years of study); consumption habits (tobacco, alcohol, drugs, medicines); family and personal medical history (including obstetric history for women, e.g. miscarriage or reproductive problems); occupational history, time of exposure to pesticides (years working and number of workdays per season each year); knowledge, training and use of safety measures at work.

In order to correlate the level of exposure with the neuropsychological and enzymes outcomes, a single variable of occupational exposure was developed that considered the number of years living in the farming area (for internal control). For the workers, the variable was constructed using years living in farming area and years working with pesticides. Because agricultural work is seasonal, workers are not always in contact with pesticides the entire year. Therefore, the amount of working years was corrected by estimating the number of days actually worked using pesticides during a calendar year. In addition, those “adjusted” years were amplified by an “occupational exposure factor” of three fold; based on publications that show Odds Ratio 3 to 7.9 of self -reported symptoms on workers after using pesticides in relation to controls in similar settings [42,43]. The equation that relates these parameters is called Days of Adjusted Life Long Occupational Exposure to Pesticides (ALLOEP) and is described as follows:

$$ \mathrm{ALLOEP} = \left(\left(\mathrm{years}\ \mathrm{living}\ \mathrm{in}\ \mathrm{agricultural}\ \mathrm{area}\ *\ 365\right) + \left(\mathrm{n}{}^{\circ}\ \mathrm{years}\ *\ \mathrm{n}{}^{\circ}\ \mathrm{days}/\mathrm{year},\ \mathrm{working}/\mathrm{contact}\ \mathrm{pesticides}*\ 3\right)\right)\ /\ 365 $$

Of course, several factors could affect the absorption of pesticides in workers and therefore exposure level would be moderated. Information about hazards, proper handling, use of protective equipment and safety measures are proven to be effective in reducing exposure in percentages from 2% up to 77%, depending on the protective equipment used [44]. The possibility of including a moderation factor to the exposure variable ALLOEP will be explored [44-47]. Refer to Table 2 to for details about variable description and factors utilized to build indicators measuring exposure.

**Table 2:** **Variable description and factors utilized to build indicators measuring exposure**

**Exposure**	**Variable description**	**Variable construction**
Environmental	Number of days living in farming area	Number of years living in farming area times 365 (days per year)
Occupational	Life-long occupational exposure (days)	Number of years living in farming area times 365 (days per year) + (number of years working in contact with pesticides times number of days working per year*)
	“Adjusted Life-long Exposure to Pesticides” - ALLOEP score	The result from the upper row amplified by 3-fold increase of exposure

(*) Number of months per year working in contact with pesticides, according to information declared in the questionnaire for seasonal workers.

### Reporting participants and feedback

Information is being distributed to stakeholders about the progress of the project on a regular basis. Individual lab results on enzymes activity and neuropsychological evaluation will be given to participants at the end of the project by the Project Director as a summary, instead of informing each individual test result from the large battery of tests.

### Epidemiologic data analysis and statistical analysis

Statistical analysis will be done in SPSS. Several steps will be followed for the data analysis, given the different specific objectives of the study.

1. To investigate the trends of the three biomarkers in each of the population groups: simple descriptions of the enzymatic activity with the support of scattering graphs are being done for each of the biomarkers in every population group (occupationally exposed, internal control group and external control), and for each one of the fumigation periods considered. ANOVA tests are being utilized to detect significant differences in the average of enzymatic activity between groups and between fumigation periods among exposed (occupational and environmental) groups. Differences on enzymatic activities within each individual will be assessed when volunteers have more than one measurement.
2. To assess risk of neuropsychological weakening in the three population groups: according to standard test scores, “normal” and “under normal” individuals were selected. Taking the external control group as a baseline to compare the performances of neuropsychological evaluation, Odds Ratio (OR) are being calculated for each of the exposed population groups (environmental and occupational exposure) and for the different fumigation seasons. In order to avoid confounding affected by age and level of education, OR are stratified by age, level of education and alcohol consumption. Chi square and Confidence Interval are being calculated for each OR in order to detect significance and power. Mantel and Heanzel correction will be used when necessary.
3. To correlate enzymatic activity of the three biomarkers with level of exposure: to reach this objective with the three population groups and different fumigation periods, we are using synthesis for exposure level in a single variable (ALLOEP).
4. To correlate enzymatic activity of the three biomarkers with neuropsychological performance: in the three population groups and different fumigation periods, test scores are being correlated with enzymatic activity for the three biomarkers.
5. To assess the performance of the activity of ACPH as a diagnostic test of prolonged exposure to OPP: ROC curves are being developed utilizing performance result in the battery of tests as gold standard. The *case definition* criteria extracted from the performance in the battery of tests are taken from the scale of each test given by the test provider.

The entire population of participants evaluated in baseline condition (pre-fumigation) were selected as “cases” and “controls” according to the “case definition criteria”; the “cases” being people with poor test performance and the “controls” being those people with normal test performance.

The contribution of enzymes or any other variables in predicting a *case* are being assessed using logistic regression model. Inclusion of other data mining techniques for exploring predictive models is currently being explored (neuronal networks and decision tree).

## Discussion

One of the strengths of this study is the assessment of more than one control group for occupational exposure, which includes the possibility of evaluating effects of environmental and occupational exposures. Inclusion and exclusion criteria improve design by avoiding selection bias and confusion (age; urban or rural social context; no neuropsychological illness, trauma and/or medication; right-handedness; no known pesticide intoxication). Additionally, all outcomes are being measured on baseline and during fumigation period, permitting the assessment of changes on enzymatic activity and neuropsychological effects of pesticides among individuals, populations, and fumigation periods. In the exposed group it is possible to assess whether biomarkers return to baseline within two to three months after cessation of exposure. Regarding the neuropsychological outcome, effects of aging will be avoided by selecting people from 18 to 50 years old; several tests will be performed and several cognitive areas explored; and we will increase the specificity of the diagnostic tool, according to suggestions described in literature [48]. Keep in mind that neuropsychological evaluation will be used as gold standard for diagnosis of cognitive impairment.

An acknowledged weakness of the study is that no result may be related to a specific chemical compound because it is not possible to identify the specific pesticides being used by the workers or determine their metabolites in either biological or environmental specimens. According to the literature [49,50], we assume that organophosphate and carbamates are the most used pesticides in the region, according to type of crop (grapes and citrus fruits) and the season of the evaluation. The study did not evaluate retired workers because the aim was to assess biomarker in workers, and not in performing consequences or causality of exposure; nevertheless, this could be a complementary result.

This study design was implemented during the 2011–2013 sample collection, neuropsychological evaluation and data collection process. So far, seasonal recruitment of participants has been a success given the difficulties found in the work place, which have been sorted-out during subsequent years by our experience gained and contacts made. The support of the municipalities and farmers’ associations has been important to avoid problems in the field. Based on our experience over these past few years, mobile laboratory use has been a success, the application of the neuropsychological evaluation of the tests battery by a single professional has been important in order to avoid bias and quality control of the laboratory procedures has been adequate as well.

## Abbreviations

ChE: Plasma cholinesterase
AChE: Acetylcholinesterase
ACPH: Acylpeptide-hydrolase
BuChE: Butyrylcholinesterase
DFP: Diisopropyl fluorophosphates
EDTA: Type of anticoagulant vacutainers
PBS: Phosphate buffer saline
DTNB: 5.5′-dithio-bis-2-nitrobenzoic acid
DTT: Dithiothreitol
AANA: N-acetyl-L-alanine *p*-nitroaniline
DMSO: Dymethylsulfoxide
ALLOEP: Days of Adjusted Life Long Occupational Exposure to Pesticides
ANOVA: Varianza analysis
OR: Odds Ratio
ROC curves: Receiver Operating Characteristic curves
